# The Prognostic Value of Hyponatremia for Predicting Poor Outcome in Patients With COVID-19: A Systematic Review and Meta-Analysis

**DOI:** 10.3389/fmed.2021.666949

**Published:** 2021-06-14

**Authors:** Mohammad Rizki Akbar, Raymond Pranata, Arief Wibowo, Irvan, Teddy Arnold Sihite, Januar Wibawa Martha

**Affiliations:** ^1^Department of Cardiology and Vascular Medicine, Faculty of Medicine Universitas Padjadjaran, Rumah Sakit Umum Pusat Hasan Sadikin, Bandung, Indonesia; ^2^Faculty of Medicine, Universitas Pelita Harapan, Tangerang, Indonesia

**Keywords:** coronavirus–COVID-19, sodium, mortality, severe, critical, hyponatremia

## Abstract

**Background:** This meta-analysis aimed to assess the prognostic value of hyponatremia in patients with COVID-19.

**Methods:** We performed a systematic literature search on PubMed, Scopus, ScienceDirect, and Wiley up until January 26, 2021. The key exposure was hyponatremia, defined as sodium level below the reference level. The outcome of interest was poor outcome, which was a composite of mortality, severe COVID-19, and prolonged hospitalization. Severe COVID-19 was defined severe CAP or needing ICU care or IMV. The pooled effect estimate was odds ratio (OR). Sensitivity, specificity, positive and negative likelihood ratio (PLR and NLR), diagnostic OR (DOR), and area under curve (AUC) were generated.

**Results:** There were 11,493 patients from eight studies included in this systematic review and meta-analysis. The incidence of hyponatremia was 24%, and incidence of poor outcome was 20%. Hyponatremia was associated with poor outcome in COVID-19 (OR 2.65 [1.89, 3.72], *p* < 0.001; I^2^: 67.2%). Meta-regression analysis showed that the association between hyponatremia and poor outcome was reduced by age (OR 0.94 [0.90, 0.98], *p* = 0.006) and hypertension (OR 0.96 [0.93, 0.94], *p* < 0.001). Hyponatremia has a sensitivity of 0.37 [0.27, 0.48], specificity of 0.82 [0.72, 0.88], PLR of 2.0 [1.5, 2.7], NLR of 0.77 [0.69, 0.87], DOR of 3 [2, 4], and AUC of 0.62 [0.58, 0.66] for predicting poor outcome. In this pooled analysis, hyponatremia has a 33% posttest probability for poor outcome, and absence of hyponatremia confers to a 16% posttest probability.

**Conclusion:** Hyponatremia was associated with poor outcome in patients with COVID-19.

**Systematic Review Registration:** PROSPERO, CRD42021233592.

## Introduction

Coronavirus disease 2019 (COVID-19) is now one of the most common diseases in the world (1). Although most of the patients have mild–moderate symptoms, a significant number of patients develop organ failure and require critical care (2). Identifying patients that require a more intense monitoring is of paramount importance due to limitation of human and medical resources, especially in the developing countries. Patients' characteristics and laboratory features proved to be helpful for this purpose (3–6).

COVID-19 may develop pneumonia and lead to sepsis that produces high levels of inflammatory cytokines (7, 8). Interleukin (IL)-1β, IL-6, and tumor necrosis factor (TNF) play a pivotal role in the development of sepsis. IL-6 has an inverse correlation with serum sodium which has a mechanism to stimulate hypothalamic arginine vasopressin (9–12). Several studies showed that pneumonia is associated with syndrome of inappropriate antidiuretic hormone (11, 13, 14); this mechanism may cause hyponatremia. Hyponatremia is related with prolonged time in the ICU and increased mortality rate in sepsis, cardiovascular disease, and chronic kidney disease (11, 15). Hyponatremia has been shown to increase mortality in patients with COVID-19. However, the prognostic value of this finding is still unclear. Several laboratory abnormalities have been shown to indicate poor prognosis; however, it does not directly translate to clinical value. This is the first systematic review and meta-analysis to address whether hyponatremia is a reliable biomarker. This meta-analysis aimed to assess the prognostic value of hyponatremia in patients with COVID-19.

## Materials and Methods

This is a Preferred Reporting Items for Systematic Reviews and Meta-Analyses (PRISMA) guidelines compliant meta-analysis. This meta-analysis is registered in PROSPERO (CRD42021233592).

### Eligibility Criteria

We included studies that fulfill all of these criteria: (1) observational retrospective and prospective studies, (2) COVID-19 patients, (3) hyponatremia and normonatremia, and (4) mortality/severe COVID-19/prolonged hospitalization/need for intensive unit care (ICU)/invasive mechanical ventilation (IMV).

We excluded the following studies: (1) preprints, (2) case reports, (3) conference abstracts, (4) review articles, and (5) abstract-only publication. Preprints were excluded to reduce bias. Conference abstracts or abstract-only publications were excluded because they often did not contain sufficient information and the analyses were only described in brief.

### Search Strategy and Study Selection

We performed a systematic literature search on PubMed, Scopus, ScienceDirect, and Wiley with keywords (“COVID-19” OR “Coronavirus Disease” OR “SARS-COV2”) AND (Hyponatremia OR Hyponatremic OR “Low Sodium”) up until January 26, 2021. We removed the duplicates and screened the title/abstract of the records. This process was performed by two independent authors, and discrepancies were resolved by discussion.

### Data Extraction

Data extraction of the included studies was performed by two authors (R.P and I.I) independently. Both authors are medical doctors experienced in conducting systematic review and meta-analysis. The data of interest includes the author, design of the study, baseline characteristics, and outcome of interest. Discrepancies were resolved by discussion.

We made unsuccessful attempts to contact authors for studies that do not sufficiently report their data required for our analysis.

### Exposure and Outcome

The key exposure was hyponatremia, defined as sodium level below the reference level for eunatremia. The outcome of interest was poor outcome, which was a composite of mortality, severe COVID-19, and prolonged hospitalization. Severe COVID-19 was defined severe pneumonia or needing ICU care or IMV. The pooled effect estimate was odds ratio (OR). Sensitivity, specificity, positive and negative likelihood ratio (PLR and NLR), diagnostic odds ratio (DOR), and area under the curve (AUC) were generated.

### Risk of Bias Assessment

Risk of bias assessment was performed by two authors independently, using the Newcastle–Ottawa Scale (NOS) tool (16). There are three domains in NOS: (1) selection (representativeness, selection of comparator, and ascertainment of exposure), (2) comparability (outcome of interest was not present at the start and the two groups were comparable), and (3) outcome (independency of outcome, adequacy of follow-up, and lost to follow-up). Discrepancies were resolved by discussion.

### Statistical Analysis

The incidence of hyponatremia and poor outcome was pooled using the meta-analysis of proportion. The random-effects DerSimonian–Laird method was used to generate the pooled effect estimate in form of OR and its 95% CI. *P*-values <0.05 were considered as statistically significant. To evaluate heterogeneity of the pooled analysis, we performed the I-squared (I^2^) and Cochran Q tests, in which a value of <50% or *p* < 0.10 indicates significant heterogeneity. Sensitivity, specificity, PLR, NLR, DOR, and AUC were generated to evaluate the prognostic value of hyponatremia. To assess small-study effects and publication bias, funnel-plot analysis and Egger's were performed. Trim-and-fill analysis using the linear L0 estimator was performed due to asymmetrical funnel plot. Restricted-maximum likelihood (REML) meta-regression was performed for the association between hyponatremia and poor outcome, using age, male (gender), hypertension, diabetes, and chronic kidney disease as covariates. We performed sensitivity analysis by removing non-cohort studies. We used STATA 16 (StataCorp) to perform meta-analysis.

## Results

**Figure 1:**
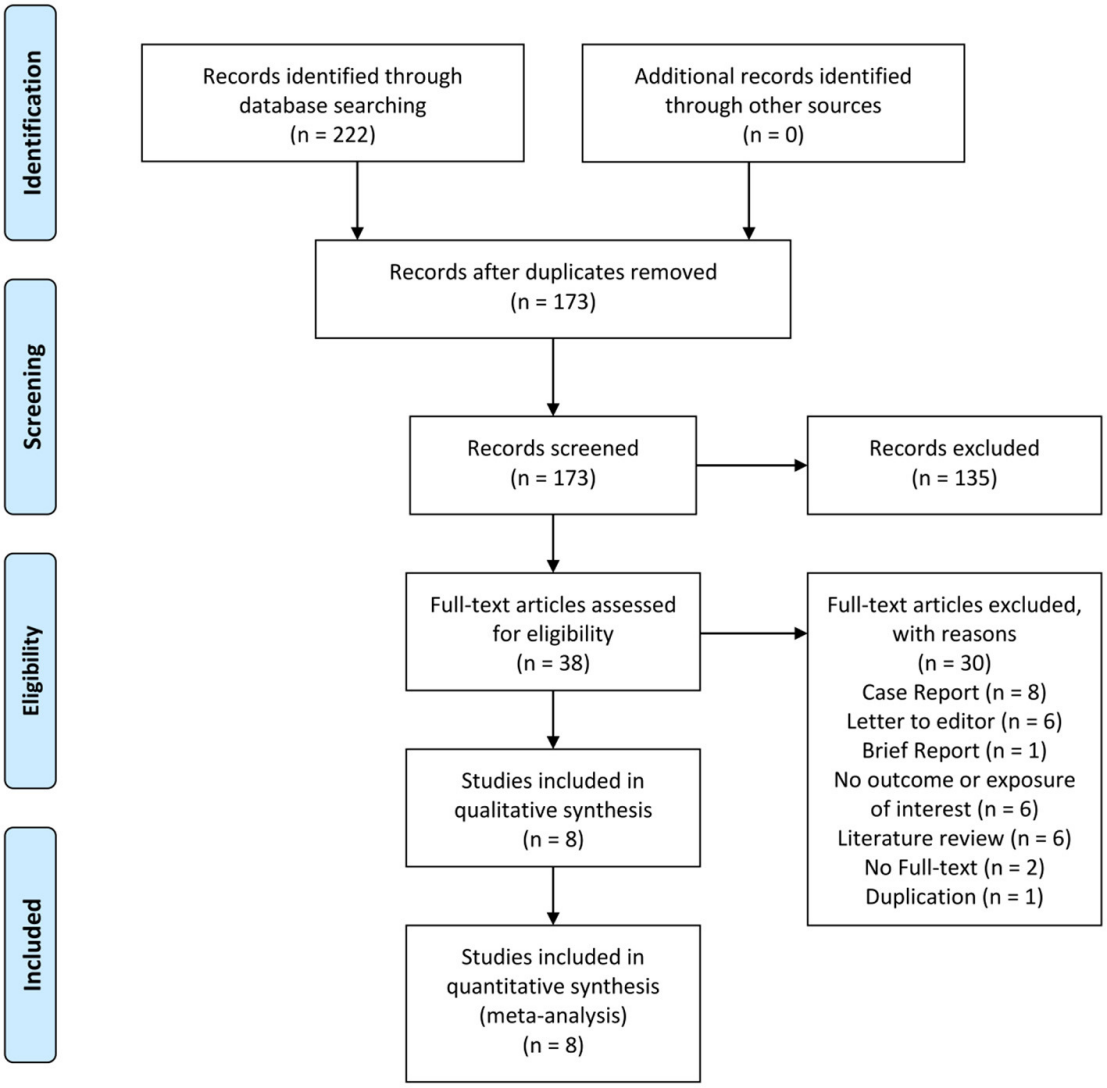
PRISMA Flowchart.

There were 11,493 patients from eight studies included in this systematic review and meta-analysis (Figure 1) (17–24). Table 1 displays baseline characteristics of the included studies. The overall risk of bias as assessed with NOS was moderate. The incidence of hyponatremia was 24% [16%−31%]. The incidence of poor outcome was 20% [14%−35%].

**Table 1:** Baseline characteristics of the included studies.

**First author**	**Design**	**Sample**	**Cutoff point** ** (meq/l)**	**Age** ** (years)**	**Male** ** (%)**	**Hypertension** ** (%)**	**Diabetes** ** (%)**	**CKD** ** (%)**	**CVD** ** (%)**	**Outcome**	**NOS**
DeCarvalho, 2021	Case–control	594	≤ 135	65	55.8	39.6	18.7	9.3	14.1	ICU	6
Fendo, 2020	Retrospective	4490	<135	65.1	58	49.1	19	6.8	23.5	Mortality	8
Frontera, 2020	Retrospective	4452	≤ 120	64	65.6	43.2	30.3	13.2	8.5	Mortality	7
Hu, 2020	Retrospective	1254	<135	56.1	51.1	27.5	14.7	3.1	5.7	Mortality	6
Sarvazad, 2020	Cross-sectional	54	121–134	56	57	NR	NR	NR	NR	ICU	6
Tezcan, 2020	Retrospective	408	<135	54.3	46	31.9	23.5	3.2	10.5	Mortality	6
Wu, 2020	Retrospective	125	<136	55	52.8	28	20	NR	8.8	Prolonged hospitalization	7
Zeng, 2020	Retrospective	147	<135	42	61.1	16.1	7.4	0.7	5.4	ICU	5

*CKD, chronic kidney disease; CVD, cardiovascular disease; ICU, intensive care unit; NOS, Newcastle–Ottawa scale; NR, not reported*.

**Figure 2:**
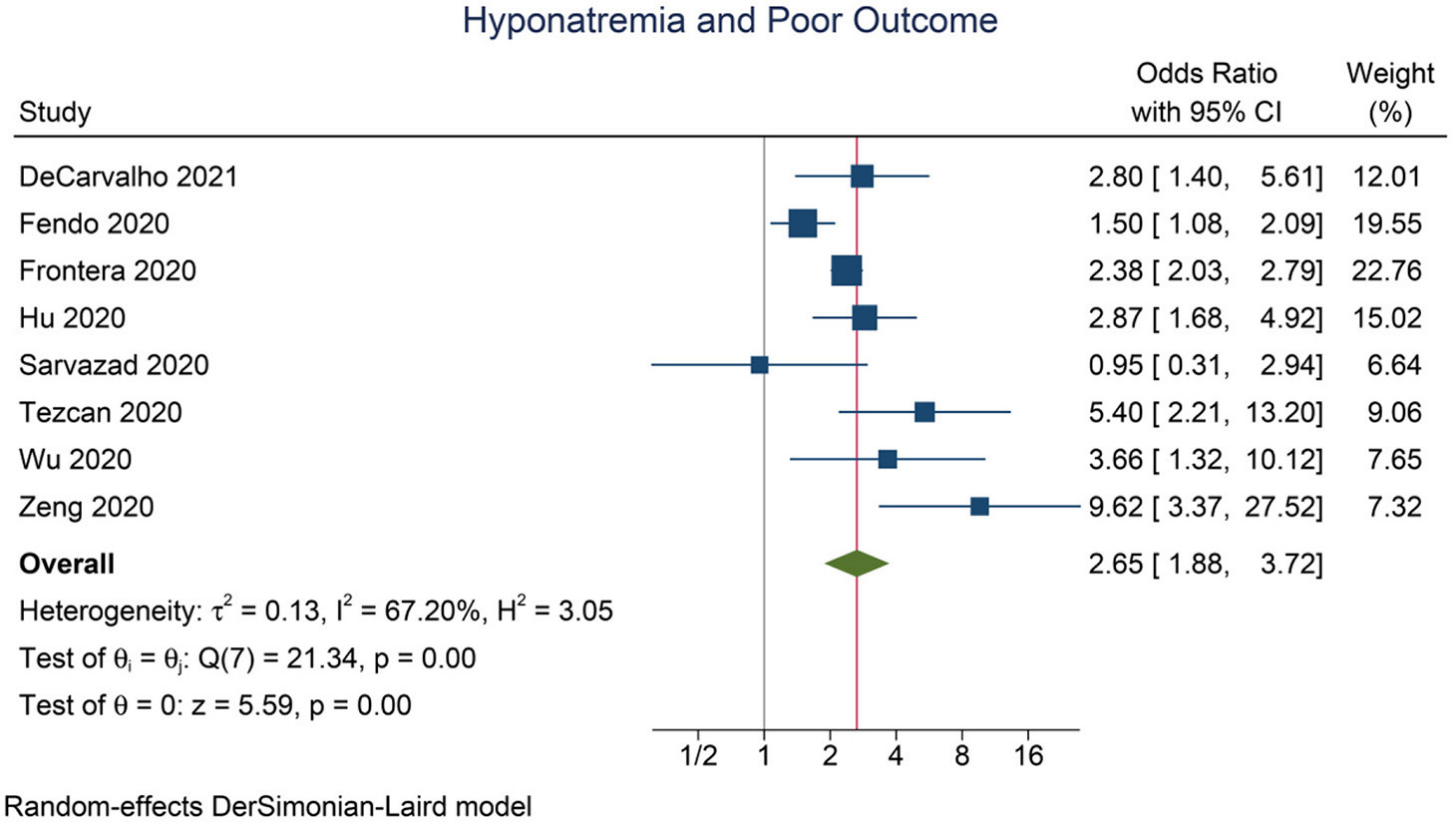
Hyponatremia and poor outcome.

**Figure 3:**
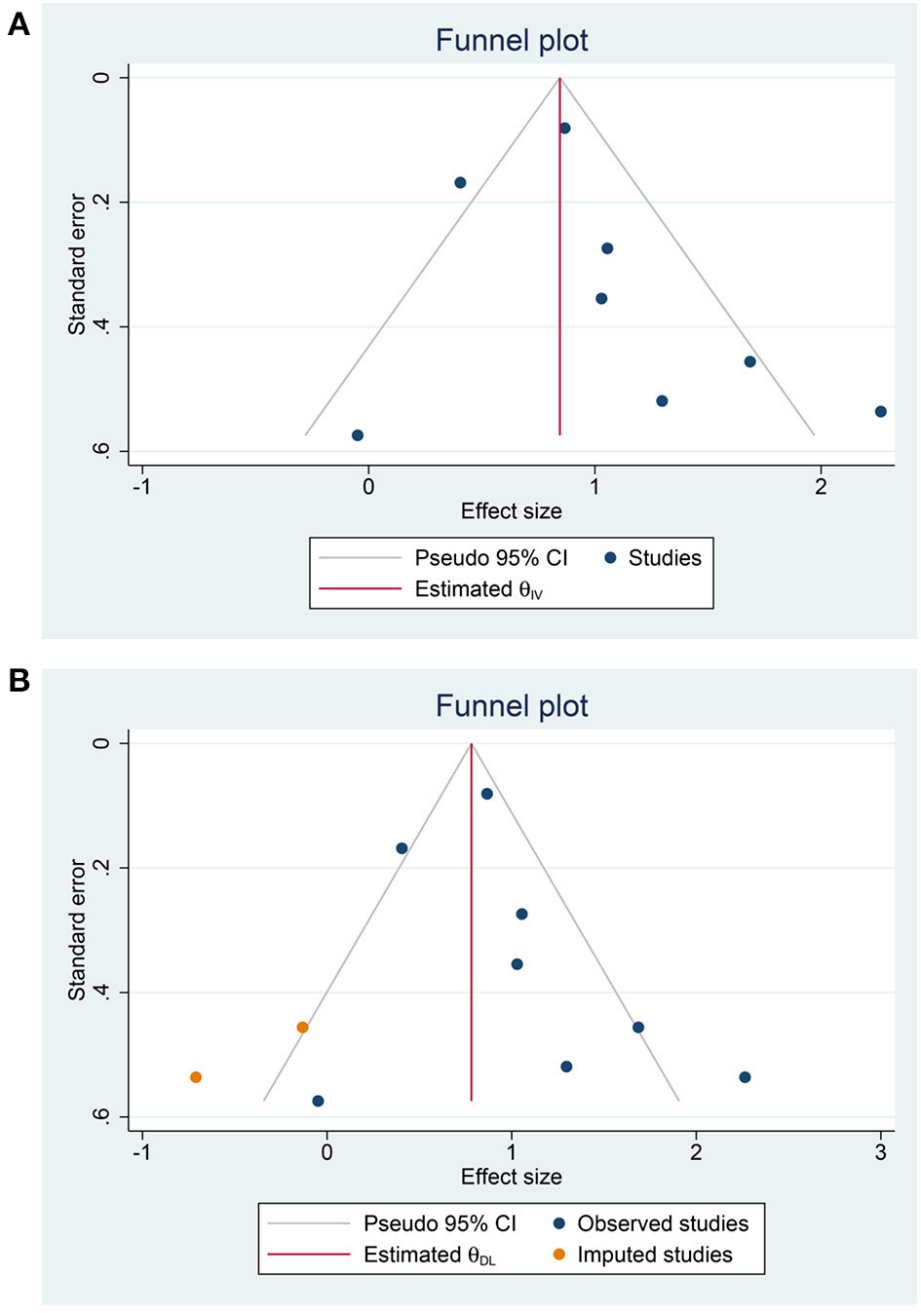
Publication bias. Funnel-plot analysis **(A)** and trim-and-fill analysis **(B)**.

Hyponatremia was associated with poor outcome in COVID-19 (OR 2.65 [1.89, 3.72], *p* < 0.001; I^2^: 67.2%, *p* = 0.003) (Figure 2). The funnel plot was asymmetrical (Figure 3A), and trim-and-fill analysis resulted in an OR of 2.65 [1.89, 3.72] (Figure 3B). There is no indication for small-study effects (*p* = 0.231). Meta-regression analysis showed that the association between hyponatremia and poor outcome was reduced by age (OR 0.94 [0.90, 0.98], *p* = 0.006) and hypertension (OR 0.96 [0.93, 0.94], *p* < 0.001), but not male (gender) (*p* = 0.532), diabetes (*p* = 0.308), and chronic kidney disease (*p* = 0.177).

**Figure 4:**
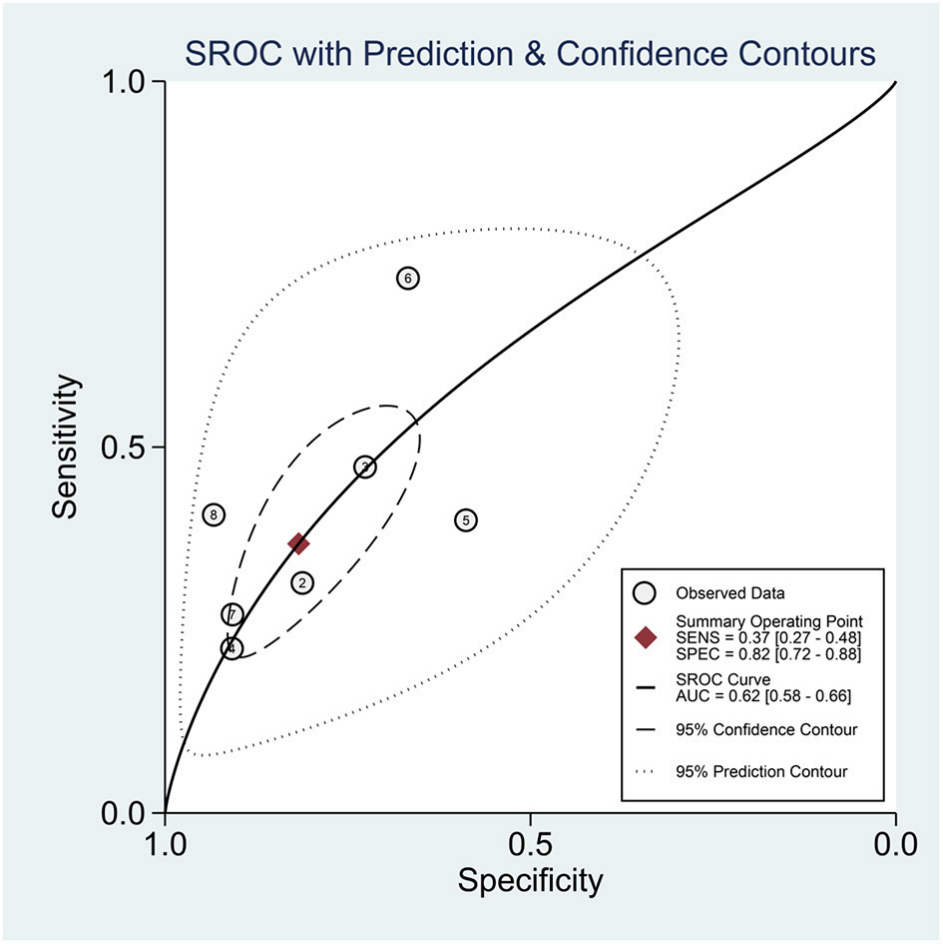
SROC curve.

**Figure 5:**
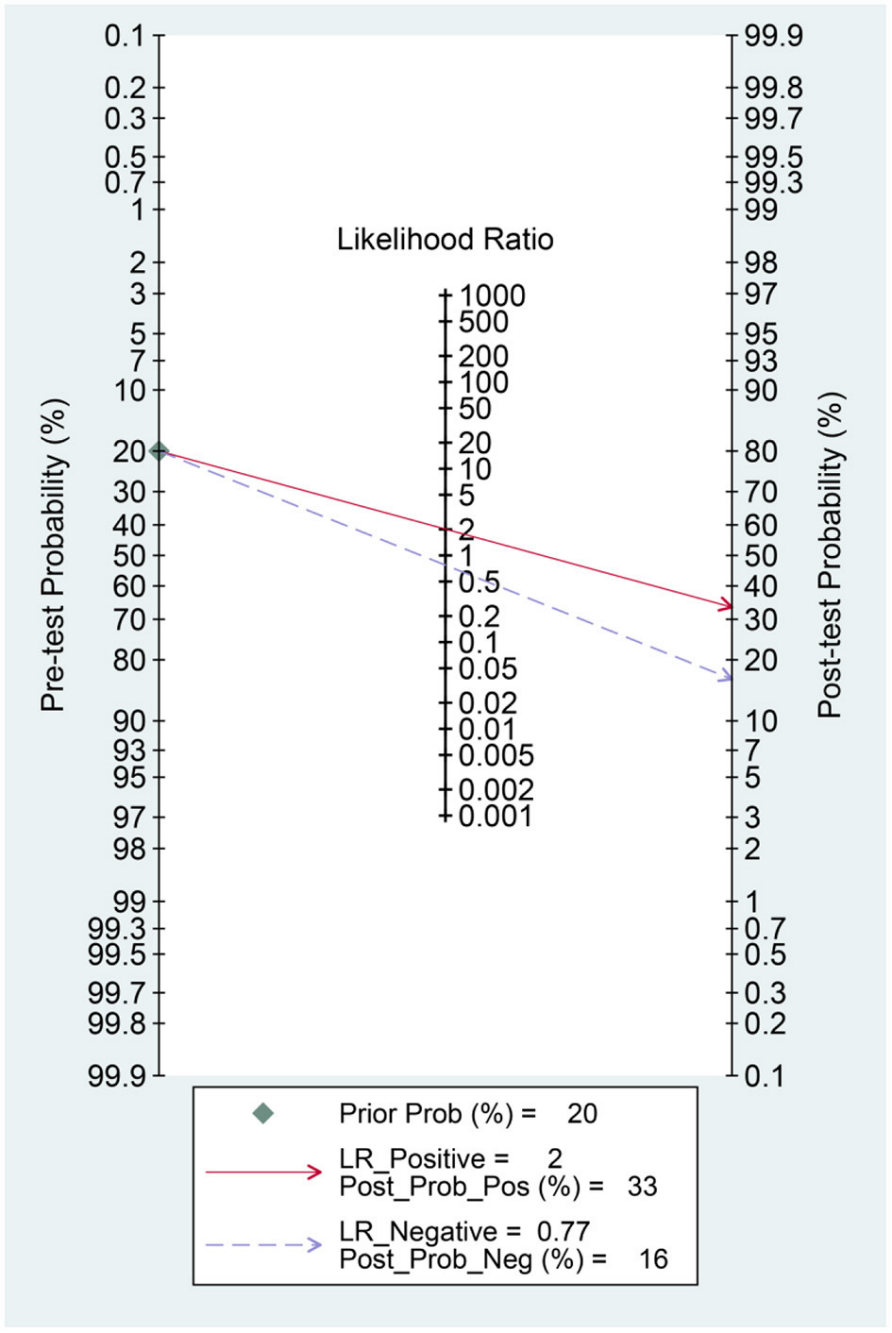
Fagan's nomogram.

**Figure 6:**
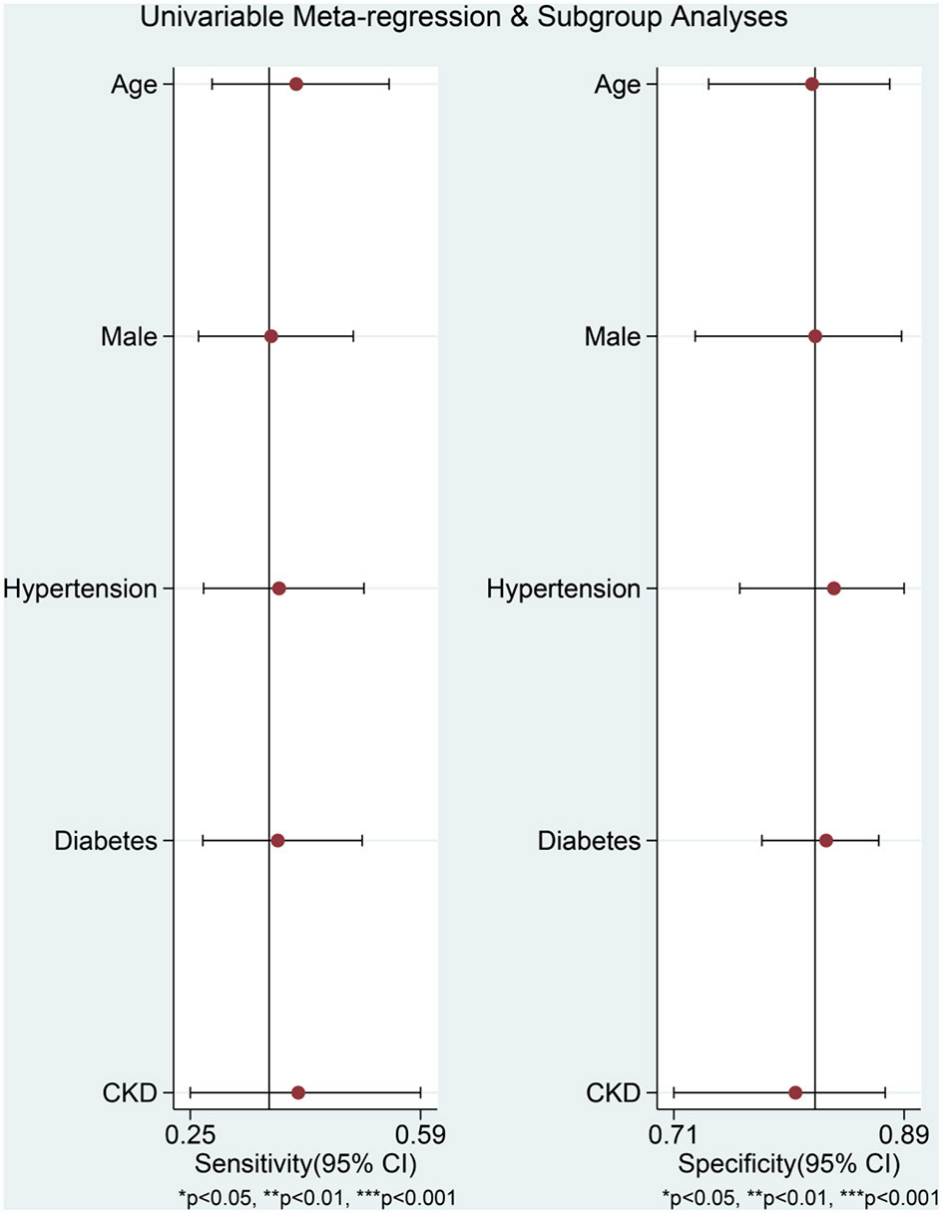
Meta-regression analysis.

Hyponatremia has a sensitivity of 0.37 [0.27, 0.48], specificity of 0.82 [0.72, 0.88], PLR of 2.0 [1.5, 2.7], NLR of 0.77 [0.69, 0.87], DOR of 3 (2, 4), and AUC of 0.62 [0.58, 0.66] for predicting poor outcome (Figure 4). In this pooled analysis, hyponatremia has a 33% posttest probability for poor outcome, and absence of hyponatremia confers to a 16% posttest probability (Figure 5). Meta-regression and subgroup analysis showed that the sensitivity and specificity did not vary by age, male (gender), hypertension, diabetes, and chronic kidney disease (Figure 6).

Sensitivity analysis by removing non-cohort studies showed that hyponatremia was associated with poor outcome (OR 2.88 [1.95, 4.27], *p* < 0.001; I^2^: 73.2%, *p* = 0.002), with sensitivity of 0.35 [0.24, 0.48], specificity of 0.84 [0.75, 0.90], PLR of 2.1 [1.5, 3.0], NLR of 0.77 [0.67, 0.87], DOR of 3 (2, 4), and AUC of 0.64 [0.60, 0.69].

## Discussion

Hyponatremia was associated with poor outcome in patients with COVID-19 with a 37% sensitivity and 82% specificity. There was an indication of publication bias as shown by the funnel-plot analysis; however, after trim-and-fill analysis, the result was still significant.

All of the studies except Sarvazad et al. demonstrate a significantly elevated risk of poor outcome in patients with hyponatremia. The study has a small sample size and contributes to the least weight in the pooled analysis. One of the possible explanations for the statistical insignificance is due to the inadequate sample size.

Meta-regression analysis showed that older age and hypertension modify the association, in terms of OR, between hyponatremia, and poor outcome. Both older age and hypertension have been shown to increase mortality in patients (25–27). The explanation for this modifying finding is currently unclear. However, neither variable significantly modifies the sensitivity and specificity.

In patients with COVID-19, volume depletion from gastrointestinal fluid losses causes ADH release that leads to hyponatremia (18). Additionally, SIADH is frequently encountered in patients with pneumonia and may contribute to hyponatremia (18, 28). IL-6 levels have been shown to be inversely correlated, and IL-6 itself has been associated with increased severity in patients with COVID-19; thus, hyponatremia may signify cytokine storm (29, 30). Additionally, hyponatremia may cause several neurological complications such as cerebral edema, seizures, and encephalopathy (22). An overly aggressive correction of hyponatremia may lead to central pontine myelinolysis; these factors may contribute to mortality and severity in patients with COVID-19.

Although this meta-analysis showed the association between hyponatremia and poor outcome in COVID-19, it did not necessarily equal to causality. Hyponatremia might be a bystander factor; for example, hyponatremia might only indicate increased IL-6 or other parameters or condition that may actually increase the risk of poor outcome. Patients with hypertension may also experience hyponatremia through diuretics use, and meta-regression analysis indicates that the association between hyponatremia and poor outcome is slightly reduced in this population. This finding may imply that hyponatremia due to the natural history of COVID-19 is more significant than hyponatremia due to other causes, although further investigation is required.

### Clinical Implications

Hyponatremia indicates a higher risk for poor outcome in patients with COVID-19; however, due to low sensitivity but high specificity, it can be used to rule in, but not rule out, poor prognosis. Moreover, hyponatremia alone has a poor AUC for predicting poor outcome in patients with COVID-19. Thus, combining hyponatremia with other variables in a prediction model will be more useful rather than using it as a standalone parameter.

### Limitations

One limitation of this meta-analysis is publication bias in which there are more positive studies compared to negative studies being published. Most of the studies were retrospective in nature and highly prone to bias. This meta-analysis contained observational studies that either were descriptive or analyze cohorts with a prognostic objective. This meta-analysis established the association between hyponatremia and poor outcome in COVID-19, but did not prove causality. Finally, this meta-analysis provides the assumption that hyponatremia should be a risk factor to be taken into account in future prognostic models that may be constructed when exploring COVID-19 risk factors.

In conclusion, hyponatremia was associated with poor outcome in patients with COVID-19.

## Data Availability Statement

The raw data supporting the conclusions of this article will be made available by the authors, without undue reservation.

## Author Contributions

MA: conceptualization, investigation, writing—review and editing, and supervision. RP: conceptualization, methodology, software, data curation, formal analysis meta-analysis, investigation, validation, writing—original draft, and writing—review and editing. AW: investigation and writing—original draft. I: data curation, investigation, and writing—original draft. TS and JM: investigation and writing—review and editing. All authors contributed to the article and approved the submitted version.

## Conflict of Interest

The authors declare that the research was conducted in the absence of any commercial or financial relationships that could be construed as a potential conflict of interest.

## Abbreviations

AUC: area under curve
COVID-19: Coronavirus disease 2019
ICU: intensive care unit
IL: interleukin
IMV: invasive mechanical ventilation
NOS: Newcastle–Ottawa Scale
NLR: negative likelihood ratio
OR: odds ratio
PLR: positive likelihood ratio
REML: restricted-maximum likelihood
SIADH: syndrome inappropriate antidiuretic hormone
TNF: tumor necrosis factor.
